# Pond dyes are *Culex* mosquito oviposition attractants

**DOI:** 10.7717/peerj.3361

**Published:** 2017-05-31

**Authors:** Natali Ortiz Perea, Amanda Callaghan

**Affiliations:** Ecology and Evolutionary Biology Section, School of Biological Sciences, University of Reading, Reading, United Kingdom

**Keywords:** Urban ecology, British mosquito, *Culex pipiens*, Ponds, Oviposition, Pond dye, Behaviour, Disease, Mosquito control, Habitat

## Abstract

**Background:**

British mosquito population distribution, abundance, species composition and potential for mosquito disease transmission are intimately linked to the physical environment. The presence of ponds and water storage can significantly increase the density of particular mosquito species in the garden. *Culex pipiens* is the mosquito most commonly found in UK gardens and a potential vector of West Nile Virus WNV, although the current risk of transmission is low. However any factors that significantly change the distribution and population of *C. pipiens* are likely to impact subsequent risk of disease transmission. Pond dyes are used to control algal growth and improve aesthetics of still water reflecting surrounding planting. However, it is well documented that females of some species of mosquito prefer to lay eggs in dark water and/or containers of different colours and we predict that dyed ponds will be attractive to *Culex* mosquitoes.

**Methods:**

Black pond dye was used in oviposition choice tests using wild-caught gravid *C. pipiens*. Larvae from wild-caught *C. pipiens* were also reared in the pond dye to determine whether it had any impact on survival. An emergence trap caught any adults that emerged from the water. Water butts (80 L) were positioned around university glasshouses and woodland and treated with black pond dye or left undyed. Weekly sampling over a six month period through summer and autumn was performed to quantified numbers of larvae and pupae in each treatment and habitat.

**Results:**

Gravid female *Culex* mosquitoes preferred to lay eggs in dyed water. This was highly significant in tests conducted under laboratory conditions and in a semi-field choice test. Despite this, survivorship in black dyed water was significantly reduced compared to undyed water. Seasonal analysis of wild larval and pupal numbers in two habitats with and without dye showed no impact of dye but a significant impact of season and habitat. Mosquitoes were more successful, with significantly higher numbers of pupae, in the habitat where they had vegetation cover and shade.

**Discussion:**

Our study has raised some interesting possibilities; one is that where used, pond dyes may be encouraging mosquitoes to breed in gardens in close proximity to people. Considering the concerns over potential future spread of disease in urban environments, this as well as shading of ponds and water butts, should inform future advice over reducing mosquito breeding and spread.

## Introduction

West Nile virus (WNV) is a positive-sense RNA virus belonging to the Flaviviridae family and is transmitted by mosquitoes, including *C. pipiens* complex mosquitoes. There have been several major outbreaks of WNV in Europe in recent years, affecting both humans and horses (Calistri et al., 2010; Di Sabatino et al., 2014; Hernández-Triana et al., 2014). A laboratory test of the vectorial competence of European *C. pipiens*, including the phenotypic and physiological variant *C. molestus*, demonstrated that both the *molestus* form and a hybrid between C. *pipiens* and C. *molestus* were capable of transmitting WNV (Brustolin et al., 2016). Both of these variants are found in Britain, raising the possibility that outbreaks of human or animal viral diseases could occur in Britain if conditions and climate permitted. Whilst the threat is likely to come from invasive species, more than 30 species of mosquito, including putative vectors of arboviruses, are native to the UK (Blagrove et al., 2016). To date there is no evidence of mosquito-borne virus transmission of public health concern in the UK (Blagrove et al., 2016). However, we know that mosquitoes are established in both rural and urban habitats and are often found in gardens (Townroe & Callaghan, 2014). Understanding and mitigating future threats requires detailed ecological knowledge of the putative vector species and prediction of how mosquito populations are influenced by anthropogenic activity.

In England, 80% of the human population lives in towns and cities which cover more than 7% of the land area (Wilby & Perry, 2006). Urbanisation changes the physical environment in a way which is known to alter habitat types, species numbers and the community composition of ecosystems (McKinney, 2006; Sala et al., 2000). These changes are likely to impact British mosquito populations and influence distributions, abundances, species composition, mosquito-host interactions, biting nuisance and the potential for mosquito borne disease to occur in the UK.

Gardens make up a large proportion of the urban area and provide a significant contribution to the green spaces within many UK cities providing areas of ecological value which may support diverse wildlife populations including mosquitoes (Smith et al., 2005). The creation of ponds is encouraged as a means of enhancing the biodiversity value of gardens, particularly in the face of a widespread decline of ponds in the wider rural landscape (Gaston et al., 2005). Although individually small (∼2.5 m^2^) and fragmented into small patches, urban ponds are distributed widely across the urban landscape and are likely to contain water all year round (Gaston et al., 2005). Where fish are not present, they are likely to provide a valuable breeding site for mosquitoes. Water butts also provide an ideal habitat for mosquitoes with a recent study recording five British mosquito species; *Anopheles claviger*, *An. plumbeus*, *Culesita annulata*, *C. pipiens* and *C. torrentium* (Townroe & Callaghan, 2014). Predicted future changes to the climate, with increased summer temperatures and more frequent heavy rainfall in winter, will continue to place pressure on water supplies and encourage domestic water storage (Snow & Medlock, 2006). This in turn is likely to increase populations of the most common species, *C. pipiens*, particularly in urban gardens.

*C. pipiens* is a potential enzootic (primary) vector of West Nile Virus—WNV (Medlock & Leach, 2015) and the species most likely to be directly affected by changes in water storage and pond formation (Townroe & Callaghan, 2014). The current risk of WNV transmission in the UK is considered low because the abundance of enzootic and bridge (non-primary) vectors is too low for sustained transmission (Medlock & Leach, 2015). Changes in climate, migration of mosquito species and longer flight seasons in dense urban areas creates conditions more conducive to high levels of human host biting and an increased risk of disease transmission. Therefore, any factors that significantly change the distribution and population of *C. pipiens* are likely to impact subsequent risk of disease transmission.

Pond dyes are a relatively new cosmetic product for garden ponds and lakes. They have proved to be popular at recent high profile garden shows such as Chelsea and Hampton Court. They stop the growth of algae by blocking the red end of the visible light spectrum (of wavelength 620–740 nm) from penetrating the water. The red end of the light spectrum is needed for photosynthesis, as peak absorption for photosynthetic pigments is approximately 650 nm (Douglas, Raven & Larkum, 2003). Although there is no evidence to suggest that these dyes are toxic to fish and invertebrates, the impact on invertebrate communities may well be behavioural. In this study, we investigate the impact that pond dyes have on oviposition and survival in *C. pipiens* mosquitoes. Previous studies have shown that the cues for oviposition are often visual and have demonstrated a preference for oviposition in dark containers and dark waters (Beckel, 1955; Collins & Blackwell, 2000; Hilburn, Willis & Seawright, 1983; Hoel et al., 2011; Panigrahi et al., 2014). We therefore predict that pond dyes will act as an attractant for mosquito ovipostion, with a potential impact to increase mosquito population densities in garden ponds. This is the first study to specifically look at pond dyes to see if they impact on mosquito breeding behaviour and success.

## Materials and Methods

### Trapping wild gravid female mosquitoes

Wild gravid female Culicine mosquitoes were trapped using modified oviposition traps (Reiter, 1987; Townroe & Callaghan, 2015). A total of 10 traps were placed on the Whiteknights campus at the University of Reading, Berkshire, England (51.4419°N, 0.9456°W). Approximately 1,000 gravid female mosquitoes were caught in July and August 2014 and 2015. Most of the mosquitoes sampled belonged to the *Culex* genus although a few *Anopheles plumbeus* (<5) and *Culiseta annulata* (<5) were also trapped.

### Oviposition preferences of wild mosquitoes

An oviposition choice experiment was performed by releasing 200 of the trapped gravid mosquitoes into a tent (245 × 145 × 95 cm) placed outdoors on campus (51.4419°N, 0.9456°W). Mosquitoes were allowed to freely oviposit in one of 14 2 L plastic containers placed randomly in the tent: seven with 1.2 L tap water and seven with 1.2 L tap water treated with pond dye at the concentrations recommended by the manufacturer (Dyofix, Leeds, UK). After seven days, the containers were taken to the laboratory to count egg batches laid in each container. The experiment was performed three times with freshly trapped females and a choice between tap water and black colour dye.

The choice experiment was repeated with wild-caught gravid females under laboratory conditions (25 °C, 16:8 light:dark). Five groups of 20 gravid females were chosen randomly and each group transferred into a cotton net cage 25 × 25 × 25 cm per treatment set. In each cage, two 200 ml plastic bowls were filled with 150 ml of either tap water or dye water. The choice experiments were repeated in normal rearing conditions (16:8 light:dark) and also in the absence of light (black bags were used as a cover in each cage during the experiment).

### Emergence study

A modified emergence trap (Hamer et al., 2011) was used to measure the impact dye had on mosquito survival. Eggs from the oviposition experiment were hatched in the laboratory (25 °C, 16:8 L:D) and reared in tap water through to 2nd instar, fed with pelleted rabbit food. One hundred were then transferred to each of 18 11 L plastic bins (23 × 28 cm) containing 10L tap water or 10L tap water and dye (Dyofix, Leeds, UK). Food was added to each bin (1.2 g guinea pig food) which was capped with a conical fabric mesh to trap emerging adults. The bins were placed outdoors in the area used to trap the females.

Traps were monitored daily for emerging adult mosquitoes. These were captured using a manual aspirator, transferred into small tubes and stored at −20 °C for identification (Snow, 1990).

### Wild population numbers in dye treated and untreated artificial containers

Thirty two 80 L water butts (44.5 cm × 58.5 cm, Townroe & Callaghan, 2014) were placed around the secure area behind the School of Biological Sciences Harborne building on Whiteknights campus in the summer of 2014. Each container was filled with 60 L of tap water and 8 g of ground oak leaves. Bins were placed in pairs with the second bin additionally containing black pond dye added according to manufacturers instructions (DyoFix, Leeds, UK). For each treatment, eight replicates were organized in each habitat: woodland (51°26′12.8″N; 0°56′39.7″W) and glasshouses (51°26′13.2″N; 0°56′31.2″W). Bins pairs were several metres apart. Containers were sampled weekly for 26 weeks in 2014. Sampling was carried out using a device adapted from Onyeka (1980) and Townroe & Callaghan (2014). The device included three sections of drain pipe (4 cm high, 0.4 cm thick and 8 cm diameter) bolted together in line with fine mesh net glued to the bottom of each ring and a flexible wire handle attached to the outer edge of the furthest two rings. The device was lowered into the container and allowed to rest on the bottom for 5 min before being drawn swiftly up through the water to collect animals. This method was carried out once per container per sampling event. The number of larvae and pupae collected were recorded and larvae were replaced in the container. All pupae were taken to the laboratory for rearing to adult then frozen at −22 °C. Adults were identified using a 10–40× magnification microscope using the key of Cranston et al., (1987).

### Statistics

All statistical analyses were performed using R version 3.2.2 (R Core Team, 2015). Data were tested for normality using a Shapiro–Wilk normality test. Where data were normally distributed, parametric statistics were used and oviposition data were analysed using a paired *t*-test and one-way analysis of variance (ANOVA). Oviposition in the tent was not normally distributed and a nonparametric Mann–Whitney *U*-test was performed. Differences in adult emergence between treatments were analysed using a generalised linear model binomial test. Abundance data in water butts were Log (*x* + 1) transformed and the relationship between mosquito abundance, treatment and location was analysed using 2 way repeated measures ANOVA.

## Results

### Mosquito oviposition selection in laboratory conditions and oviposition selection in the tent

**Figure 1:**
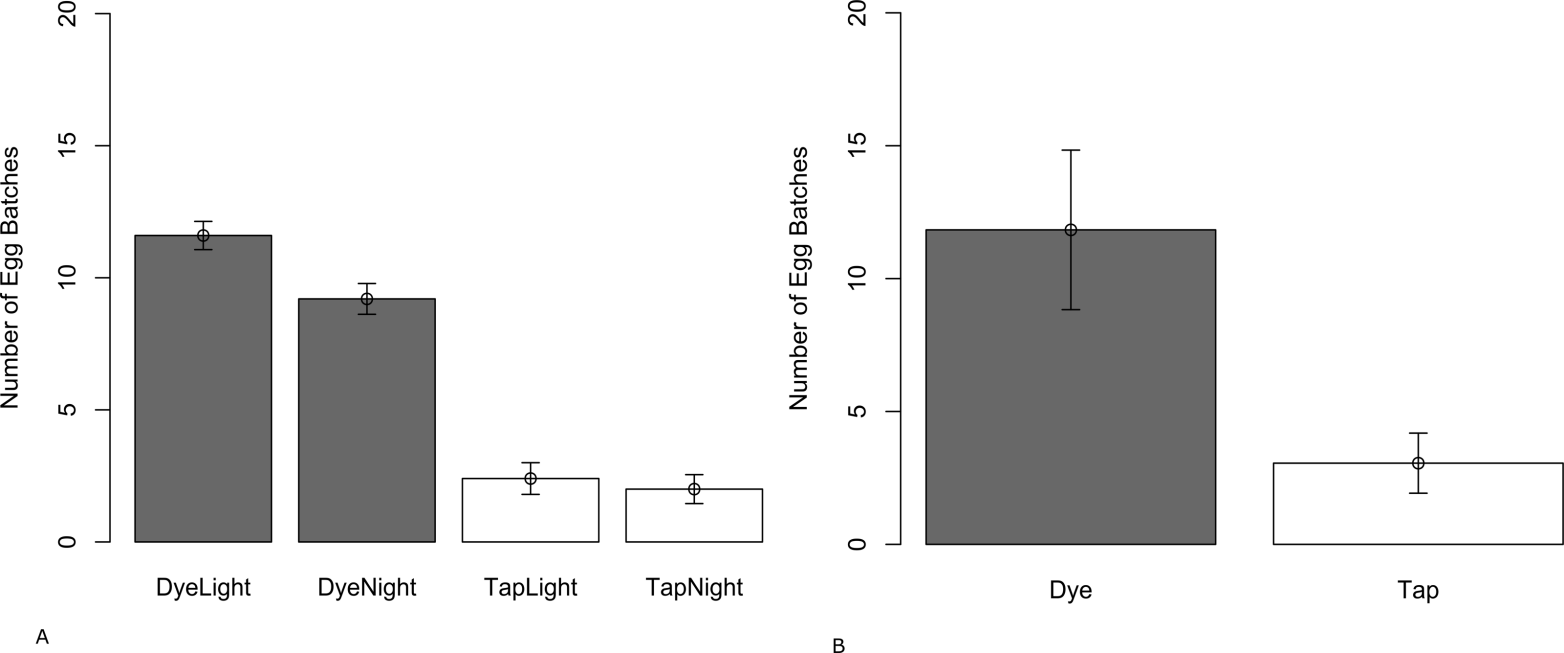
Mean number of egg batches (±SE) laid by wild-caught *C. pipiens* in paired choice tests in (A) the laboratory with a 16:8 Light/Dark plus or minus a blackout cover and (B) semi-field conditions (tent).

In the laboratory experiment, wild gravid females laid significantly more egg rafts in dye water compared with tap water (*t* = 5.4928; *df* = 8; *P* < 0.001) (Fig. 1A). Similar results were observed in the tent; wild gravid females laid significantly more egg rafts in the dye water compared to the tap water (*W* = 250; *P* < 0.001) (Fig. 1B). Females laid significantly fewer eggs in the dye treatment when there was a reduction in light (*t* = 3.0358; *df* = 8; *P* = 0.016). Light had no significant effect on numbers of eggs laid in tap water (*t* = 0.49237; *df* = 8; *P* = 0.6357). Even though females laid fewer eggs when light was reduced, they still preferred to lay in dye water rather than tap water (*t* = 9; *df* = 8; *P* ≤ 0.001).

**Figure 2:**
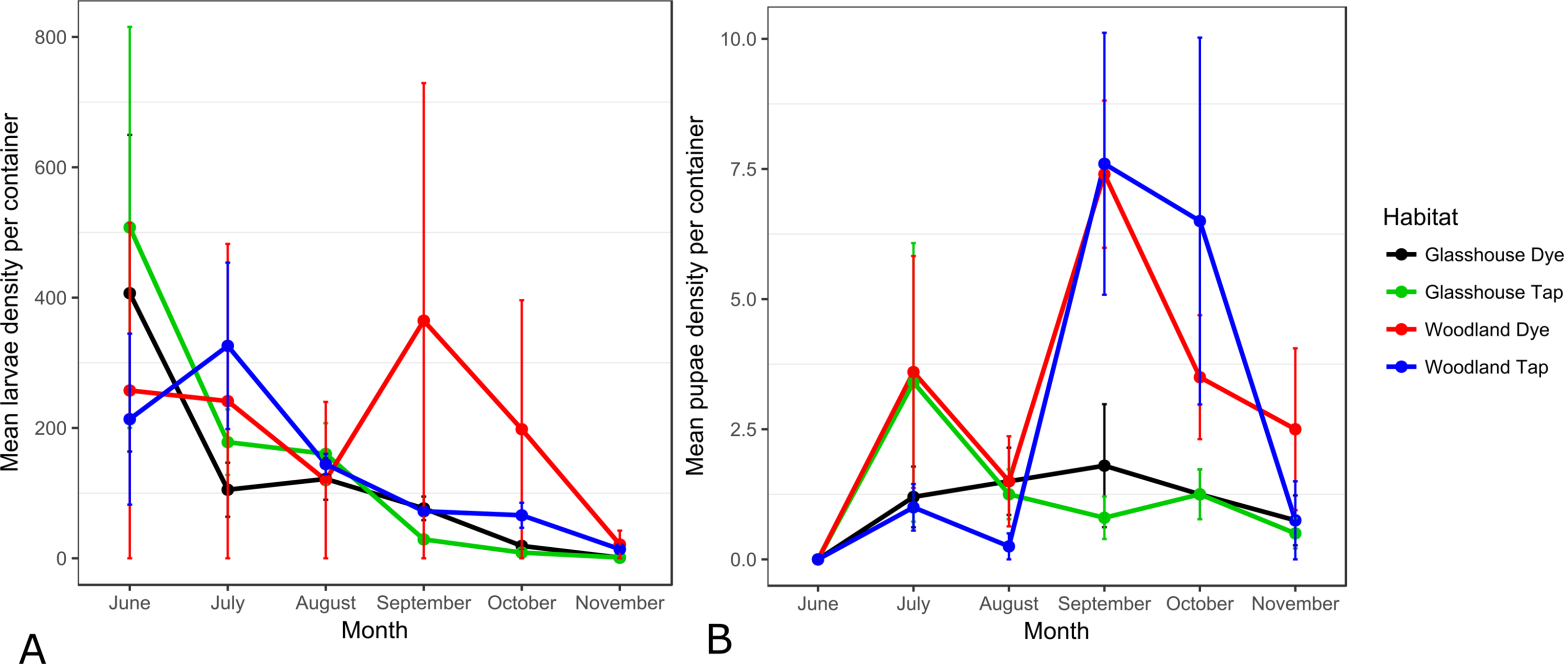
Mean (±SEM) number of *C. pipiens* larvae (A) and pupae (B) sampled in dye and tap water in woodland and glasshouse habitats.

### Wild population numbers in dye treated and untreated artificial containers

Larval and pupal numbers were analysed by season; summer (June–August) and Autumn (September–November), treatment and habitat (Fig. 2). No significant differences were observed in larval or pupal densities between dye and tap water in the summer (larval *F*_124_ = 0.062; *p* = 0.8048; pupal *F*_124_ = 0.034; *p* = 0.856) or in the autumn (larval *F*_124_ = 0.162; *p* = 0.691; pupal *F*_124_ = 0.002; *p* = 0.962). Habitat impacted on larval numbers, with higher numbers in the glasshouse in the summer (*F*_124_ = 4.488; *p* = 0.045) and higher numbers of pupae in the woodland in the autumn (*F*_124_ = 4.240; *p* = 0.049). However, there were no habitat differences in larval densities in the autumn (*F*_124_ = 0.130; *p* = 0.722) or in pupal densities in the summer (*F*_124_ = 0.002; *p* = 0.969).

**Figure 3:**
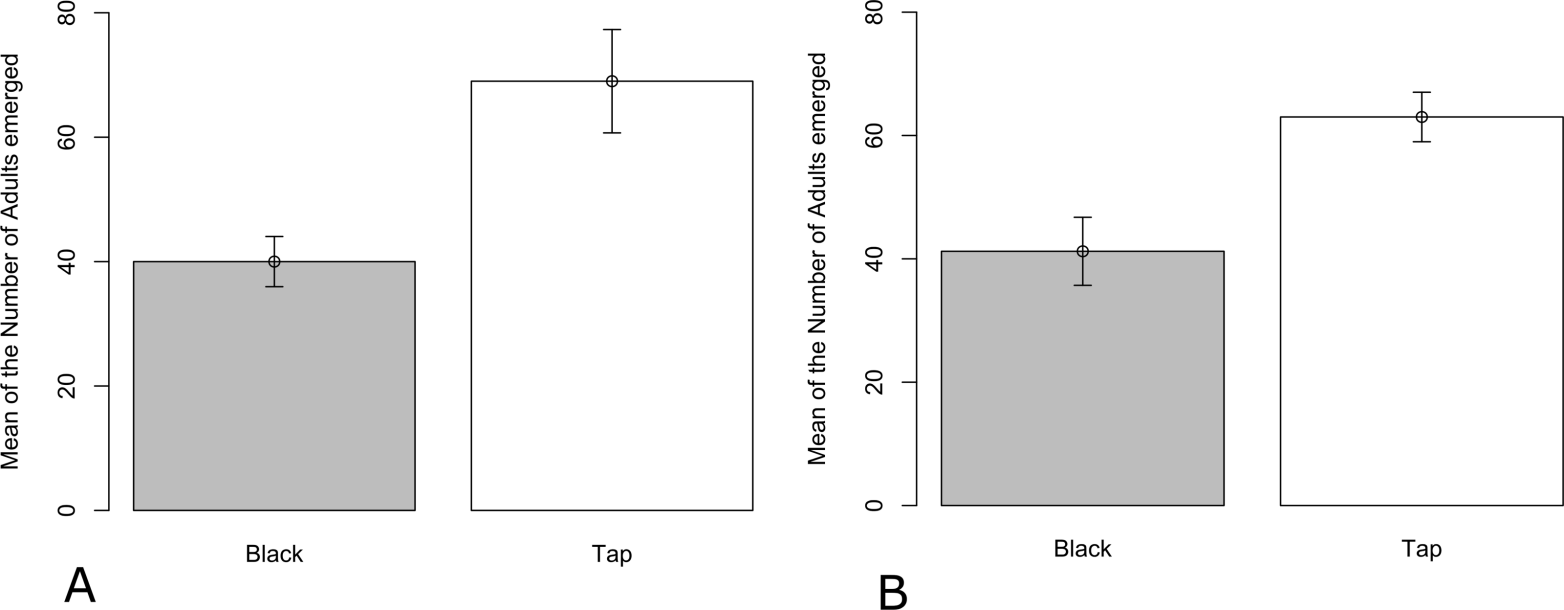
Mean number (±SEM) of *C. pipiens* adults emerging from bins containing tap water or tap water and dye in (A) Summer and (B) Autumn.

### Emergence study: field conditions

The total number of adults emerging from different treatments varied significantly in both summer (*Z* =  − 11.800, *P* < 0.001) and autumn (*Z* =  − 9.172; *P* < 0.001) (Fig. 3). In each season, fewer adults emerged from the black dye.

## Discussion

Urban garden ponds represent an abundant and reliable network of aquatic resources within which juvenile mosquitoes may develop and adults move across the landscape. Adding pond dyes might influence the attractiveness of ponds as breeding sites to mosquitoes. This is important since the exploitation of human domestic habitats has facilitated mosquito-borne human disease outbreaks in other parts of the world, such as the WNV outbreaks in North America (Patz et al., 2004).

It was not unexpected to find that *C. pipiens* females prefer to lay eggs in water with the black dye. It is well known that female mosquitoes have preferences for oviposition in containers of different colours and previous studies have demonstrated oviposition choice in dyed water although no work has been undertaken on pond dyes (Collins & Blackwell, 2000; Li et al., 2009; Oliva, Correia & Albuquerque, 2014; Beehler & DeFoliart, 1990; Beehler, Millar & Mulla, 1993; Isoe et al., 1995). A possible explanation for this is that mosquitoes choose to oviposit in dark containers as it indicates depth and therefore a lower threat of desiccation before juveniles develop. It might also indicate a higher concentration of organic matter providing nutrition (Hoel et al., 2011; Williams, 1962). Another suggestion is that the dark water mimics shading of the water body (Vezzani et al., 2005).

Visual cues seem to have some importance. Covering the adult cages with black plastic in the laboratory oviposition experiment significantly reduced oviposition in black dye containers, although oviposition remained significantly higher than in the control suggesting that either some light was leaking in or that other factors were in play.

Although the black dye was an oviposition attractant, it had a significant negative impact on the survival of mosquitoes through to adults. Adult mosquitoes still emerged from the dye-treated water but the breeding success of the female was almost halved by the low survivorship. The results of the breeding experiment were repeated with the same significant reduction in emergence in dyed water. Laboratory tests have found no evidence of acute toxicity of dyes to *Culex* larvae over 48 h that would explain this result. The poor survival of mosquitoes is therefore unlikely to be related to dye toxicity. It is also unrelated to the algal-killing property of the dye. If mosquitoes were in a natural environment where algae were a significant part of their diet, we might hypothesise that dye would impact survival by killing the algae. However in this artificial system larvae were given a supply of food and no algae were present in either treatment.

It is well known that mosquito larvae and pupae dive in the water column in response to threat, relying on visual or mechanical cues (Awasthi, Wu & Hwang, 2012). This requires considerable amounts of energy and constant or deep diving is associated with increased mortality (Lucas et al., 2001). It is pure speculation to suggest that the dye changes the behaviour of *C. pipiens* but in fourth-instar *Anopheles gambiae* growing in murky water columns deep diving increased significantly compared to clear water columns (Tuno et al., 2004).

Monitoring of wild population numbers in dye treated and untreated artificial containers were undertaken in two habitat types. The greenhouse habitat represented one in full sun where undyed water would reflect light presenting a large contrast between water treatments and the woodland habitat would have potentially less of a contrast since there was a lower light level.

Habitat type was found to be far more important than dye in determining the number of larvae and pupae, with the darker woodland habitat producing significantly more pupae compared to those in the brighter greenhouse area and the greenhouse habitat producing significantly more larvae. There is little information on the impact of shade on British mosquitoes but this result agrees with that of Fischer & Schweigmann (2004), who found that seasonal patterns of abundance of *C. pipiens* in urban Argentina showed positive relationships with vegetation cover. A further study on container breeding mosquitoes in an Argentinian cemetery found that the numbers of both *C. pipiens* and *Aedes aegypti* immatures were higher in shaded containers than in containers in full sun (Vezzani & Albicocco, 2009; Vezzani et al., 2005). Clearly it is likely that temperatures were higher in Argentina and shaded, cooler, containers have higher adult mosquito production rates because of a negative effect of high temperatures (Vezzani et al., 2005). In our study the greenhouse habitat produced significantly more larvae, although this did not translate into more pupae, possibly indicating larval mortality.

The fact that pond dye treatment had no impact on wild mosquito numbers can be explained by two possibilities. One is a balancing of oviposition preference against survival. If more eggs are laid and yet fewer mosquitoes emerge because of the dye, the net effect could well be neutral in terms of numbers of mosquitoes produced by the habitat. The second is that although pond dye is an important factor for *Culex* female oviposition in artificial environments, there are many factors in play that will influence the success of mosquitoes in a natural habitat, including temperature and shading.

We undertook the wild population experiment using water butts rather than ponds for two reasons. The first was to limit the number of factors that might interfere with the experiment such as competing mosquito species and predators to allow us to determine whether dye was an important factor in mosquito breeding success in a more natural setting. The second is that water butts are an important urban habitat for *C. pipiens* mosquitoes and an estimated 60% of UK garden water butts are colonised (Townroe & Callaghan, 2014). Our results demonstrate that the dyes do influence both mosquito behaviour and survival but there is no evidence that this translates into a significant difference in mosquito numbers.

Populations of *C. pipiens* are expected to increase with future changes to the landscape and climate, and it has been suggested that towns and cities represent some of the highest risk areas for potential transmission of bird-related mosquito-borne disease (Snow & Medlock, 2006). The ornithophagic habit of *C. pipiens* limits its potential as a bridge vector but seasonal abundance and other eco-behavioural characteristics predispose this species to serve as a potential enzootic vector of WNV, capable of maintaining cycles among bird populations, in the UK (Medlock, Snow & Leach, 2005). It is important to understand environmental factors that might impact on mosquito population success in urban habitats, particularly if these factors are anthropological in nature.

##  Supplemental Information

10.7717/peerj.3361/supp-1 Supplemental Information 1 Oviposition choice experiment of wild caught gravid Culex with and without dye. Emergence of Culex from dye treated and untreated water

10.7717/peerj.3361/supp-2 Supplemental Information 2 Seasonal sampling of Culex larvae and pupae in dye treated and untreated water butts in the glasshouse area

10.7717/peerj.3361/supp-3 Supplemental Information 3 Seasonal sampling of Culex larvae and pupae in dye treated and untreated water butts in the woodland area

## Additional Information and Declarations

### Data Availability

The following information was supplied regarding data availability: The raw data has been supplied as a Supplementary File.
